# Supplementary material for: Assessment of a 4-Week Starch- and Sucrose-Reduced Diet and Its Effects on Gastrointestinal Symptoms and Inflammatory Parameters among Patients with Irritable Bowel Syndrome
Source: Nutrients. 2021 Jan 28;13(2):416. doi: 10.3390/nu13020416 (PMC7911460; doi:10.3390/nu13020416)

Supplemental material

Supplemental tables and figures

Table S1. Recommendations of fruit intake according to a starch- and sucrose- reduced diet

**Well tolerated**	**Tolerated by some**	**Not tolerated**
Avocado	Persimmons	Apples
Blackberries	Plums	Apricots
Blueberries	Raisins	Bananas
Boysenberries	Watermelon	Cantaloupe
Cherries		Dates
Cranberries		Grapefruit
Currants		Guava
Figs		Honeydew melon
Gooseberries		Mangos
Grapes		Nectarines
Kiwi		Oranges
Lemons		Peaches
Limes		Pineapple
Loganberries		Tangelos
Olives		Tangerines
Papaya		
Pears		
Pomegranates		
Prunes		
Raspberries		
Rhubarb		
Strawberries		

Supplemental material

Table S2. Recommendations of vegetable and legume intake according to a starch- and sucrose-reduced diet

Well tolerated	Tolerated by some	Not tolerated
Alfalfa sprouts	Edamame soybeans	Beets
Artichokes*	Jicamas	Black beans
Arugulas	Leeks	Black-eyed peas
Asparagus*	Okra	Butternut
Bamboo shoots	Pumpkin	Carrots
Bok choy	Snow peas	Cassava
Broccoli*	Tempeh	Chickpeas
Brussel sprouts*	Tofu	Corn
Cabbage*	Yellow wax beans	Garlic
Cauliflower*		Green peas
Celery		Lentils
Chard		Kidney beans
Chicories		Lima beans
Chives		Navy beans
Collard greens		Onion
Cress		Parsnips
Cucumber		Pinto beans
Eggplant		Potatoes
Endive		Soybeans
Green beans		Split peas
Kale		Sweet potatoes
Lettuce		Yams
Mung bean sprouts		
Mushrooms		
Mustard green		
Peppers		
Radishes		
Spaghetti squash		
Spinach		
Tomatoes		
Turnips		
Yellow squash		
Zucchini		

*excess intake can cause bloating/flatulence in all individuals

Supplemental material

Table S3. Participant characteristics and physical activity at baseline

	**Intervention N=80***		**Control N=25****		***p***
	**Median (IQR)**	**No of participants (%)**	**Median (IQR)**	**No of participants (%)**	
BMI (kg/m^2)	24.7 (22.3-29.1)		24.0 (22.4-26.6)		0.23
Sex (female/male)		60/20		22/3	0.14
Physical activity per week					0.44
None		9 (12)		2 (8)	
<30 min		19 (24)		5 (20)	
30-60 min		12 (13)		4 (16)	
60-120 min		20 (26)		6 (24)	
>120 min		18 (23)		8 (32)	
Subgroups					0.26
IBS-D		23 (30)		3 (12)	
IBS-M		29 (36)		8 (32)	
IBS-C		13 (17)		7 (28)	
Unspecified IBS		2 (3)		1 (4)	
Non-IBS FGID		11 (14)		6 (24)	

IBS = irritable bowel syndrome, IBS-D = diarrhea-predominant IBS, IBS-M = mixed IBS, IBS-C = constipation-dominated IBS, FGID = functional gastrointestinal disease. IBS subgroup diagnosis based on Rome IV criteria [22]. BMI: 7 missing values (mv) for the intervention group and 2 mv for controls. IBS subgroups: 2 mv for the intervention group. Mann-Whitney U test and Fisher´s exact test. $P < 0.05$ was considered statistically significant.

Supplemental material

Table S4. Comorbidity and drug treatment in the IBS patients

	**Intervention N=80**	**Control N=25**	***p***
Diseases			
Allergy	11	6	0.23
Hypothyroid disease	8	4	0.47
Asthma bronchialis	8	3	0.72
Depression	9	2	1.00
Hypertension	7	3	0.70
Migraine	7	0	0.19
Drug treatment			
Antidepressants	13	5	0.43
Levaxine	9	4	0.50
Laxatives	10	2	0.42
Proton pump inhibitor	9	3	0.58
Vitamin D	7	4	0.25
Hormonal treatment	7	2	0.64
Statins	7	1	0.39
Asthma inhalators	3	3	0.14
Folic acid	4	2	0.44
Cobalamin	3	2	0.34

The number of patients with irritable bowel syndrome (IBS) with concomitant disease and regular drug treatment during the study period. One patient could have several drugs within the same category. Fisher´s exact test. $P < 0.05$ was considered statistically significant.

Supplemental material

Table S5. Vitamin intake at baseline and at the end of the 4 week-SSRD intervention

	Intervention N=80*		Control N=25**			Intervention	Control	
	Median (IQR)	% of patients ≥AR	Median (IQR)	% of patients ≥AR	*p*	Median of difference (IQR)	Median of difference (IQR)	*p*
Vitamin A (µg)						-11 (-41-28)	-1.5 (-24-32)	0.53
Baseline	510 (334-798)		383 (262-541)		0.14			
4 weeks	443 (275-709)		438 (251-646)		0.67			
Vitamin D (µg)						0.7 (-0.9-4.1)	1.9 (-0.2-3.1)	0.46
Baseline	3.5 (1.7-5.9)	16	2.4 (1.8-4.1)	4	0.13			
4 weeks	4.7 (2.2-9.8)	30	4.0 (2.8-5.7)	12	0.50			
Vitamin E (mg)						36 (-40-111)	3 (-54-86)	0.21
Baseline	9.7 (6.6-14)		9.0 (5.6-13)		0.30			
4 weeks	13 (8.8-21)		10 (6.2-14)		0.06			
Thiamine (mg)						-0.1 (-0.4-0.5)	0 (-0.5-0.2)	0.52
Baseline	1.0 (0.8-1.3)	63	0.9 (0.6-1.3)	52	0.18			
4 weeks	1.0 (0.6-1.6)	41	0.8 (0.6-1.2)	36	0.26			
Riboflavin (mg)						0.1 (-0.3-0.5)	0.1 (-0.2-0.3)	0.84
Baseline	1.3 (1.0-1.5)	55	1.0 (0.8-1.5)	36	0.09			
4 weeks	1.4 (1.1-1.8)	61	1.2 (0.8-1.6)	44	0.10			
Niacin (mg)						-1.0 (-8.2-9.0)	-2.7 (-5.7-6.5)	0.90
Baseline	16 (11-24)	63	14 (10-20)	64	0.31			
4 weeks	17 (11-25)	63	14 (11-23)	52	0.51			
Vitamin B6 (mg)						-0.1 (-0.6-0.5)	0 (-0.4-0.4)	0.79
Baseline	1.5 (1.2-2.0)	75	1.4 (0.9-1.9)	64	0.20			
4 weeks	1.6 (1.0-2.0)	66	1.4 (1.1-2.1)	68	0.75			
Folate (µg)						44 (-71.5-126)	33 (-63-97)	0.67
Baseline	226 (171-296)	61	238 (148-279)	56	0.52			
4 weeks	282 (193-417)	66	229 (131-351)	52	0.15			
Vitamin B12 (µg)						0.7 (-1.2-3.4)	0 (-0.6-1.7)	0.36
Baseline	3.1 (2.1-4.9)	90	2.4 (1.6-4.4)	84	0.17			
4 weeks	4.2 (2.8-6.9)	88	3.0 (2.1-4.5)	76	0.018			
Vitamin C (mg)						22 (-42-76)	0.5 (-26-23)	0.26
Baseline	69 (28-106)	63	54 (24-74)	52	0.20			
4 weeks	88 (31-144)	59	47 (22-98)	40	0.024			

SSRD = starch- and sucrose-reduced diet. *2 missing values (mv) at baseline and 6 mv at week 4. **3 mv at 4 weeks. AR = average requirement [21]. Nutrient levels were calculated from a single day (day 2) of 4-day food diary registrations; before and at the end of the 4-week dietary intervention. Calculations were performed with the AIVO Diet computer program [20]. Values are presented as median and interquartile ranges (IQR). Comparisons between groups were performed by Mann-Whitney U test. $P < 0.05$ was considered statistically significant.

Supplemental material

Table S6. Mineral intake at baseline and at the end of the 4-week SSRD intervention

	Intervention N=80*		Control N=25**			Intervention	Control	
Variable	Median (IQR)	% of AR	Median (IQR)	% of AI	*p*	Median of difference (IQR)	Median of difference (IQR)	*p*
Sodium (mg)						-551 (-1463-240)	-650 (-1467-456)	0.83
Baseline	2419 (1886-3153)		2205 (1911-3282)		0.96			
4 weeks	1866 (1215-2768)		2187 (1266-2650)		0.63			
Potassium (mg)						-161 (-854-802)	45 (-907-514)	0.73
Baseline	2506 (2048-3243)		2435 (1782-3183)		0.83			
4 weeks	2561 (1760-3168)		2466 (1715-3204)		0.80			
Phosphorus (mg)						88 (-304-451)	-14 (-228-168)	0.31
Baseline	1134 (941-1456)	96	981 (815-1386)	100	0.17			
4 weeks	1301 (1017-1586)	93	1082 (821-1391)	88	0.08			
Calcium (mg)						-20 (-272-307)	-59 (-352-173)	0.61
Baseline	759 (500-954)	74	685 (450-964)	68	0.72			
4 weeks	796 (552-1021)	76	783 (386-1120)	60	0.64			
Iron (mg)						-0.3 (-4.4-3.2)	-0.3 (-1.4-1.8)	0.37
Baseline	8.2 (6.1-11)	51	7.6 (4.6-9.0)	32	0.10			
4 weeks	7.5 (5.8-10)	45	7.1 (5.5-9.3)	32	0.62			
Magnesium (mg)						9 (-102-111)	-2 (-79-71)	0.99
Baseline	261 (203-354)		253 (202-325)		0.40			
4 weeks	284 (201-422)		280 (184-378)		0.56			
Zinc (mg)						0.9 (-1.8-2.9)	0.4 (-0.9-2)	0.74
Baseline	8.0 (6.3-9.9)	86	6.5 (5.4-9.1)	80	0.049			
4 weeks	8.9 (6.8-11)	81	7.5 (6.0-10)	80	0.08			
Iodine (μg)						-4 (-57-63)	5.5 (-56-28)	0.87
Baseline	103 (77-143)	50	97 (53-141)	44	0.51			
4 weeks	120 (72-157)	53	106 (83-138)	52	0.62			
Selenium (μg)						11 (-7.6-32)	4.5 (-7.2-21)	0.35
Baseline	34 (22-54)	56	27 (18-40)	40	0.16			
4 weeks	47 (33-71)	71	33 (22-50)	52	0.026			

SSRD = starch- and sucrose-reduced diet. *2 missing values (mv) at baseline and 6 mv at week 4. **3 mv at 4 weeks. AR = average intake [21]. AR for postmenopausal women were applied for all women > 50 years old. Nutrient levels were calculated from a single day (day 2) of 4-day food diary registrations; before and at the end of the 4-week dietary intervention. Calculations were performed with the AIVO Diet computer program [20]. Values are presented as median and interquartile ranges (IQR). Comparisons between groups were performed by Mann-Whitney U test. *P* < 0.05 was considered statistically significant.

Supplemental material

Table S7. Nutrient intakes and BMI in IBS and non-IBS controls

	**IBS patients N=105**	**Non-IBS controls N=105**	***p***
	**Median (IQR)**	**Median (IQR)**	
BMI (kg/m^2)	24.5 (22.4-27.7)	22.6 (19.8-26.2)	<0.001
Total energy (kJ)	6730 (5583-8658)	8005 (6573-10 034)	0.002
Nutrient intake (E%)			
Carbohydrates	43 (38-50)	46 (42-50)	0.15
Total Fat	35 (29-42)	37 (33-41)	0.24
Protein	16 (13-19)	18 (15-20)	0.017
Starch	19 (13-24)	27 (23-32)	<0.001
Monosaccharides	5.1 (3.5-7.7)	5.8 (4.1-7.2)	0.26
Disaccharides	7.9 (6.3-11)	11 (8.7-15)	<0.001

IBS = irritable bowel-syndrome, BMI = body mass index, kJ = kilojoule, E% = energy percentage. Missing values (mv) for energy intake and nutrients: 2 for IBS patients and 33 for non-IBS controls. BMI: 9 mv for IBS patients and 3 mv for non-IBS controls. Calculations were performed with the AIVO Diet computer program for IBS patients [20] and as previously described for controls from the Malmö Offspring Study [18]. Mann-Whitney U test. $P < 0.05$ was considered statistically significant.

Table S8. Correlations between changes in gastrointestinal symptoms and nutrient levels

**Change in variable (Δ-value)**	**Total IBS-SSS**	**Bloating and flatulence**	**Intestinal symptoms´ influence on daily life**
**Sucrose**	rs=0.225<br>p=0.029	rs=0.131<br>p=0.21	rs=0.148<br>p=0.15
**Monosaccharides**	rs =0.110<br>p =0.29	rs=0.024<br>p=0.82	rs=-0.009<br>p=0.93
**Disaccharides**	rs =0.291<br>p = 0.005	rs=0.182<br>p=0.08	rs=0.224<br>p=0.029
**Total sugar**	rs =0.216<br>p =0.036	rs=0.088<br>p=0.39	rs=0.104<br>p=0.32
**Carbohydrates**	r=0.123<br>p=0.001	rs=0.219<br>p=0.033	rs=0.250<br>p=0.014
**Starch**	r=0.063<br>p=0.001	rs=0.204<br>p=0.047	rs=0.152<br>p=0.14
**Protein**	r=-0.104<br>p=0.32	r=0.011<br>p=0.92	rs=-0.081<br>p=0.44
**Fat**	r=-0.083<br>p=0.43	r=0.001<br>p=0.10	rs=0.024<br>p=0.82
**Fiber**	r=0.134<br>p=0.20	r=0.107<br>p=0.30	rs=0.080<br>p=0.44

N = 97 (with one missing value for all correlations). IBS-SSS = irritable bowel syndrome-symptom severity scale. For ‘Bloating and flatulence’ and ‘Intestinal symptoms´ influence on daily life’ correlations there was one additional missing value for each, and for ‘Total IBS-SSS’ there were 2 additional missing values. Individual gastrointestinal (GI) symptoms were measured in mm on the visual analog scale for irritable bowel syndrome (VAS-IBS) [24]. Nutrient levels were calculated from a single day (day 2) of 4-day food diary registrations; before and at the end of the 4-week dietary intervention. Calculations were performed with the AIVO Diet computer program [20]. Pearson´s or Spearman´s correlation test. $P < 0.05$ was considered statistically significant.

Supplemental material

Supplemental figure S1. Flow chart over inclusion and exclusion criteria

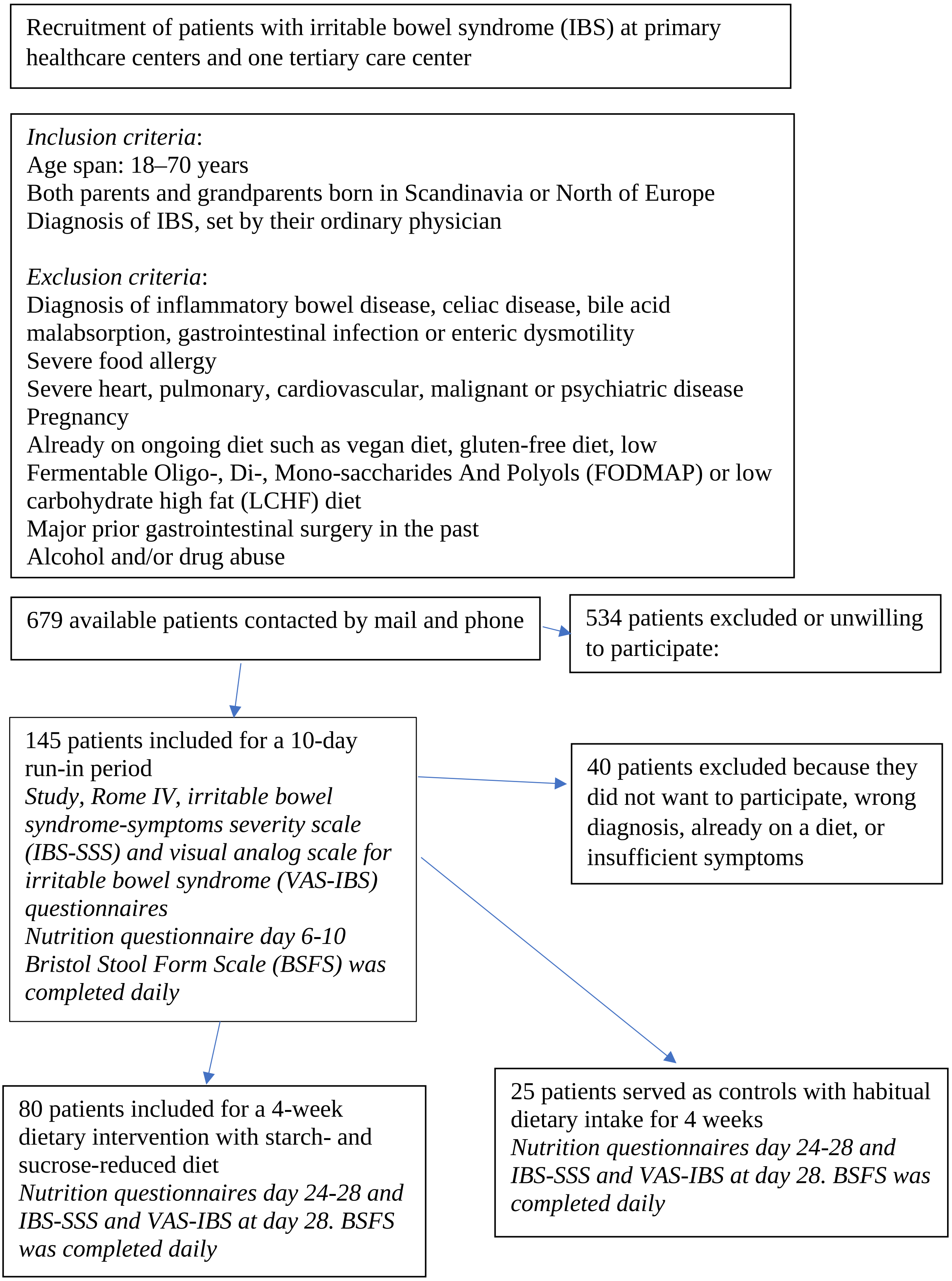

Supplement: Supplementary file 1 [file nutrients-13-00416-s001.pdf]
